# Alumni Association of Chicago Medical College

**Published:** 1867-04

**Authors:** Frank W. Reilly

**Affiliations:** Sec’y Meeting of Organization


### ALUMNI ASSOCIATION OF CHICAGO MEDICAL COLLEGE.

The following correspondence explains itself.—[Ed.

S. A. McWilliams, M.D., *Secretary Alumni Association Chicago Medical College.*

My Dear Doctor: Herewith I have the honor of transmitting you the official reports of the preliminary meeting, and of the meeting of organization, of the *Alumni Association of the Chicago Medical College*, together with the Constitution and By-Laws of the *Association* as adopted.

I am, Doctor, Your Obed't Serv't,

FRANK W. REILLY, M.D.

71 Hanover Street, *March 7th, 1867.*

### MINUTES OF A MEETING OF GRADUATES OF THE CHICAGO MEDICAL COLLEGE, HELD AT DR. DAVIS' OFFICE, SATURDAY EVENING, MARCH 2D, 1867.

Present—Drs. J. S. Jewell and John M. Woodworth, Faculty Chicago Medical College; Drs. N. W. Webber, John Quirk, Hiram Wanzer, Robert S. Addison, Julien S. Sherman, and Frank W. Reilly.

Dr. Jewell briefly explained the object of the meeting to be the organization of the Alumni of the Chicago Medical College into an association for the encouragement of goodfellowship and mutual benefit, the cultivation of a closer intimacy and stronger bonds between the common children of one Alma Mater, and, generally, the advancement of our professional interests by a formal union, holding regular meetings, correspondence and the other usual agencies of organized bodies.

On motion of Dr. Reilly, Dr. Jewell was called to preside over the meeting, and, on Dr. Woodworth's motion, Dr. Reilly was requested to act as Secretary.

On taking the chair, Dr. Jewell stated that the views and suggestions of members on the plan of organization, its aim and scope would be in order, and, after a general and informal discussion, Dr. Addison moved a committee be appointed by the Chair to draft a Constitution and By-Laws.

The Chairman appointed Drs. Woodworth, Webber, and Sherman, with instructions to be prepared to report their action at an adjourned meeting.

Dr. Jewell presented a draft of Constitution and By-Laws, which, after a reading, was placed in the hands of the above committee.

Dr. Jewell called Dr. Sherman to the chair.

The Secretary read, by request, the draft of a Constitution and By-Laws prepared by Dr. Woodworth, which draft was also referred to the committee.

On motion of Dr. Woodworth, Dr. Jewell was added to the committee on Constitution and By-Laws.

On motion, the meeting adjourned to meet in the upper lecture-room at the close of the commencement exercises on Tuesday, March 5th.

FRANK W. REILLY, M.D., *Secretary.*

---

MINUTES OF THE MEETING OF ORGANIZATION.

CHICAGO MEDICAL COLLEGE,
*Tuesday, March 5th, 1867.*

Present—Drs. Jewell and Woodworth, of the Faculty; Drs. Sherman, Webber, Merriman, Quirk, McWilliams, Reilly, Sheffield, Martin, Reckard, Robertson, Patton, Park, Parmenter, Didlake, Crocker, Oggel, Palmer, Nichols, Fredigke, Resseguie, Buchtel, Twining, Bond, Baker, Hussey, Lane, Eppler, Hutchinson, Bobb.

Dr. JEWELL called the meeting to order; the Secretary read the minutes of the previous meeting, which were adopted, and the committee on Constitution and By-Laws submitted the following:—

CONSTITUTION AND BY-LAWS OF THE ALUMNI ASSOCIATION OF THE CHICAGO MEDICAL COLLEGE.

CONSTITUTION.

I. This association shall be known as the "Alumni Association of the Chicago Medical College," and include, as members, all ordinary graduates of said college who shall make application for such purpose to the Secretary in writing, and pay the usual fees to the Treasurer.

All persons who have received the *honorary* or *ad eundem* degree shall be known as associate members, and shall be entitled to all the privileges of the association, except the right of voting and holding office.

II. The objects of this association shall be to keep alive and perpetuate that kindly and cordial feeling which binds us together by reason of our common *Alma Mater;* to encourage the interchange of professional experience, and keep alive that ardor among those who are identified with their *Alma Mater* in attempting to elevate the standard of medical education; and likewise to secure to the institution a record of the professional history of its alumni.

To this end, each member will be expected to address a letter to the Secretary on or before the 15th day of February of each year, giving a short history of his professional experience during the preceding year; and stating at length anything of special interest that may have come under his observation.

III. The officers of this association shall be a President and two Vice-Presidents, a Secretary and Treasurer. The officers shall be chosen by ballot, at each regular annual meeting, and shall hold office until their successors are duly elected.

IV. Any member may be expelled for cause, or be removed from office, after due hearing, by a two-thirds vote of the members present, at any regular meeting.

V. Every proposition to alter or amend the constitution shall be submitted in writing; a two-thirds vote of the members present being necessary to the adoption of such measure.

BY-LAWS.

I. The regular annual meeting of this association shall be held on the first Monday in March of each year. Special meetings may be called by the President, or by a Vice-President in the absence of the President.

II. The President shall preside at all the meetings of the association, and call such special meetings as he may deem necessary, or as he may be requested to call by any five members. Ten members shall constitute a quorum.

III. The Vice-President shall perform the duties of the President in case of his absence.

IV. The Secretary shall keep the records of the association; shall keep a correct list of the members, together with the date of their graduation, and Post-Office addresses. He shall also have charge of the correspondence, and shall make a report, at the annual meeting, of all communications received by him. He shall likewise collect and act as custodian of all catalogues, pamphlets, etc., relating to the history of the Chicago Medical College, and preserve copies of such original contributions to medical knowledge as may be contributed by members of the association.

V. The Treasurer shall have charge of the funds, and pay all bills authorized by a written order of the President, or, in case of his absence, by one of the Vice-Presidents; and shall render a written report at each annual meeting.

VI. Every member shall pay into the hands of the Treasurer the sum of one dollar annually.

VII. The order of business at meetings shall be as follows:—

Calling roll of members.

Reading minutes of previous meeting.

Report of committees.

Correspondence.

Deferred business.

New business.

Reading and discussion of papers.

Address.

VIII. The By-Laws may be altered or amended in the same manner as provided for in the Constitution.

The Constitution and By-Laws were unanimously adopted, the committee discharged, and, on motion, the meeting resolved itself into the ALUMNI ASSOCIATION OF THE CHICAGO MEDICAL COLLEGE.

The Chairman appointed a committee, consisting of Drs. Woodworth, Merriman, and Sheffield, to nominate officers, which committee, after a brief conference, presented the following nominations :—

*President*—J. S. JEWELL, M.D., Professor of Anatomy Chi. Med. College.

*1st Vice-President*—S. L. FULLER. M.D., Appleton, Wis.

*2d Vice-President*—D. J. HUSSEY, M.D., Cherry Valley, Ill.

*Secretary*—S. A. McWILLIAMS, M.D., Chicago.

*Treasurer*—JULIEN S. SHERMAN, M.D., Chicago.

On being duly balloted for the above gentlemen were unanimously elected, and the President, on taking his seat, made a brief address.

On motion, it was ordered that the proceedings of the preliminary meetings, together with the Constitution and By-Laws, be furnished the *Chicago Medical Examiner* for publication; and the Secretary was further instructed to issue a formal invitation, through the *Examiner*, to all graduates of the Chicago Medical College, to become members of the *Alumni Association.*

The President announced that the Constitution and By-Laws would be found at the residence of Dr. Davis, in the evening, awaiting the signatures of members; and the Treasurer announced his readiness to receive the dues of members forthwith.

Adjourned. FRANK W. REILLY, M.D.,
*Sec'y Meeting of Organization.*

